# Women and Warfare

**Published:** 1900-05-12

**Authors:** 


The Hospital

A Journal of

The Medical Sciences and Hospital Administration.

Vol. XXVIII.—No. 711.] Edited by Sir Henry Burdett, K.C.B., and Solomon C. Smith, M.D., M.R.C.P. [May 12, 1900.

### Women and Warfare.

The general, and, on the whole, the desirable tendency of modern female education among the more cultivated classes of society has been to emancipate women from a great number of the restraints which were submitted to by their grandmothers, as well as from a supposed necessity to feel and display fear in the absence of danger. It is said, indeed, that a party of lady politicians, recently assembled for the purpose of discussing the proceedings necessary for the attainment of Parliamentary franchise by their sex, was broken up in tumultuous and admired disorder by the accidental discovery of the presence, not of a man, but of a mouse. Even if this were so (and it may possibly be a mere invention of the enemy), it is at least certain that women have largely emancipated themselves from the disabilities which were at one time imposed upon them by reason of the supposed "sensibility" of the sex, and of the unwillingness of its members either to witness or to inflict suffering. Many ladies in country houses are now quite accustomed to "walk with the guns"; some have acquired sufficient dexterity to furnish their proportion of the wounded birds which are left to perish by lingering starvation; and a few have become famous as skilful and successful followers even of "big" game. In these circumstances, it is not surprising that primitive instincts, long repressed by what was perhaps, in some respects, a spurious and artificial form of civilisation, should be called into activity under the stimulus of war; and that many of our countrywomen should seek to bring themselves as nearly as possible into touch with the strife in which their brothers and their cousins are engaged. The great majority, of course, are prevented from gratifying such a wish, even if they have formed it, by the pressure of home duties, or by the want either of sufficient pecuniary means or of sufficient independence of domestic control; and hence what has been described as the recent rush of women to South Africa has been chiefly supplied by the more leisured and the more recently wealthy of the community. Women of these classes, who have never had an aim in life beyond the wearing of fine clothes and the pursuit of frivolous amusements, and who are too remote from that distinctively English type, the "great lady," to be subject to the influences of good taste or of convention, have gone out in considerable numbers, and, finding time hang heavy upon their hands, have tried to frame some justification for their presence by making a pretence of "nursing" the sick and wounded. In this undertaking, if all accounts be true, they have done much to earn for themselves the commendation which Lord Byron, in an anticipatory epitaph on himself, bestowed upon his Italian physician, Dr. Romanelli:

> "Youth, Nature, and relenting Jove,
> To keep my lamp *in* strongly strove;
> But Romanelli was so stout,
> He beat all three—and *blew* it *out*."

We hear of surreptitious gifts of currant buns to patients with enteric fever, and of the setting wide of innumerable doors for the entrance of pathogenetic bacteria; and we cannot wonder at the loud and exceeding bitter cry which these proceedings have evoked from Mr. Treves, who, happily, alike for himself and for the public, is not constrained to keep silence, either by military discipline, or by any fear of injury to a practice which rests upon too assured a basis to be disturbed. It is quite certain that his weighty words were uttered after due deliberation, and that they neither apply, nor were intended to apply, to nursing of a kind which could by any possibility be beneficial, whether this were undertaken by wealthy women, or by those to whom it would be a means of livelihood. He pointed to a definite blot in the organisation of the military hospitals—a blot which is calculated seriously to impair their efficiency, and which ought without delay to be removed. The meddling of untrained women with the nursing of cases of serious wounds or illnesses is a grave matter. Such women not only cannot do any good, but they cannot by any possibility escape from doing serious harm. If it be true, as clearly asserted in a letter from a trained nurse at the front which appeared in the *Daily Graphic* for the 5th instant, that the War Office only authorises a limited number of "nurses" in hospitals and on transports, and that this limited number is partly composed of amateurs destitute of training or knowledge, to the exclusion of competent persons, the responsibility lying on the shoulders of those in authority is, indeed, a heavy one. So far as our information goes, however, the greatest care has been taken to ensure that all Army nurses are fully trained, and we can only suppose that some confusion has arisen between the various types of hospitals at present in South Africa. It is the plain duty of the War Office to see that none but trained nurses are permitted to hold office in the hospitals, and to support the medical staff in the rigid exclusion of superfluous visitors from the wards.

